# Early recurrence of mandibular torus following surgical resection: A case report

**DOI:** 10.1016/j.ijscr.2021.105942

**Published:** 2021-04-30

**Authors:** Rabuel Valentin, Levasseur Julie, Zwetyenga Narcisse, Gengler Charline, Moris Vivien, Guillier David

**Affiliations:** Department of Maxillofacial and Oral Surgery, University Hospital of Burgundy, 2 boulevard du Maréchal de Lattre de Tassigny, BP 77908, 21079 Dijon cedex, France

**Keywords:** Torus, Exostoses, Oral surgery, Case report

## Abstract

**Introduction and importance:**

Tori are benign bony outgrowths that occur in different locations along the mandible and maxilla. Their origin is still uncertain; however, various hypotheses have been put forward, including male gender or mechanical overload. Recurrence of a torus after surgical resection is rarely described, and even less rapidly after a procedure.

**Case presentation:**

We present here the case of a 52-year-old patient who presented voluminous mandibular tori on the lingual side. The tori recurred very rapidly after the first resection surgery and with the same initial volume. Pathological examinations confirmed the histological type. The identified risk factors were excessive fish consumption, occlusal overload factors, and male gender. The patient then underwent a second surgery associated with a mouth guard in order to treat bruxism. There was no recurrence after one year of follow-up.

**Clinical discussion:**

This case report highlights the fact that there is still a lack of understanding of the risk factors associated with torus. However, several studies have been able to understand certain genetic or dietary mechanisms in the genesis of these exostoses.

**Conclusion:**

This case emphasizes the importance of mechanical overload in the recurrence of exostoses, which, coupled with dietary, gender, and ethnic factors, may be responsible for recurrence in this patient. The detection of factors associated with the risk of recurrence is a major challenge.

## Highlights

• The various factors that cause torus are still poorly understood.
• Recurrence of tori after surgery has been described only rarely, but never rapidly.
• The detection of factors associated with the risk of recurrence are a major challenge, in order to avoid recidivism.

## 1 Introduction

Tori are benign, often asymptomatic bone growths [[1], [2], [3], [4]] with commonly slow clinical evolution [1,7]. In the mouth, the most common locations are the mandibular and palatal areas. A palatal torus is generally located at the longitudinal crest of the hard palate, while a mandibular torus is located on the mandibular lingual side, above the mylo-hyoid line, next to the premolar sector, and bilaterally in 80% of cases [3,4]. These bone outgrowths are often discovered incidentally during a control examination, most often in the 3rd decade of life for palatal tori and in the 4th decade for mandibular tori [5]. The precise origins of these exostoses are uncertain, even if some radio-anatomical studies suggested genetic and environmental correlations in the development of tori [6,8]. Recurrence of a torus after surgical resection is rarely described, and even less so rapidly after the procedure.

This Case report has been written in line with the 2020 SCARE Criteria [9].

## 2 Case report

**Fig. 1:**
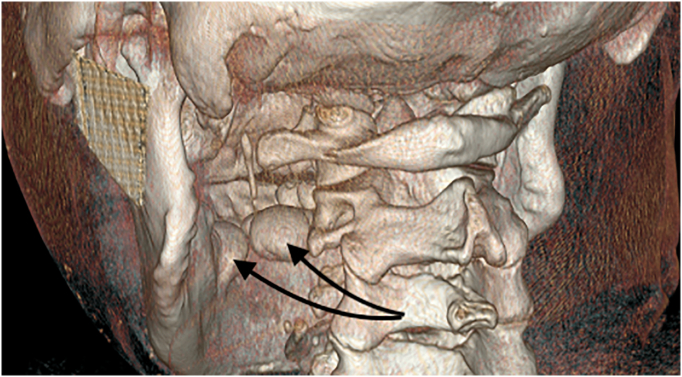
3D facial scan of the patient with posterior view.

A 50-year-old male patient was referred to the department of oral and maxillofacial surgery for a voluminous mandibular and median palatal exostosis [Fig. 1]. The patient, who was from Ivory Coast, had no medico-surgical history, treatment, allergies or history of tobacco use. There was no specific family history, in particular no history of oral exostoses. He reported frequent consumption of saltwater fish.

Clinical examination revealed signs of bruxism, with the presence of marked linea alba, significant dental attrition (Stage III according to the Rozencweig classification), and hypertrophy of the masseter muscles. The palatal torus was small in diameter (0.4 cm) and was not a cause of discomfort. The mandibular tori were voluminous (1.67 and 1.16 cm) and bothered the patient, especially in the lingual position. There were signs of mucosal inflammation due to constant rubbing of the tongue on the exostoses. A surgical resection was indicated due to functional discomfort and the results of the CT scan.

**Fig. 2:**
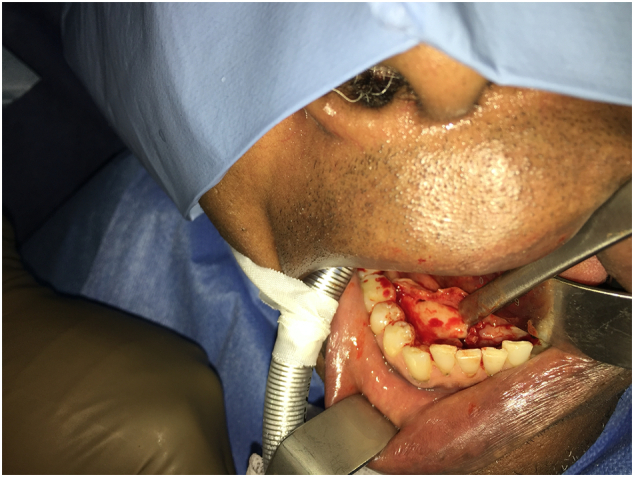
Intraoperative view of the mandibular torus.

The surgical procedure, carried out in September 2018, by the maxillofacial surgeon consisted of an excision of the mandibular tori by subtractive osteoplasty after an intrasulcular incision and detachment at the level of the base of the teeth [Fig. 2]. The full thickness flap was raised to the base of the tori bilaterally.

Removal of the tori was performed with piezotome® under 0.9% saline irrigation and separation with bone scissors. Bone regularization was performed with a round burr until a smooth and regular mandibular lingual slope was obtained. *Re*-application of the flaps was performed by suturing with Fast Vicryl® 4/0. Antibiotic prophylaxis was administered intraoperatively.

**Fig. 3:**
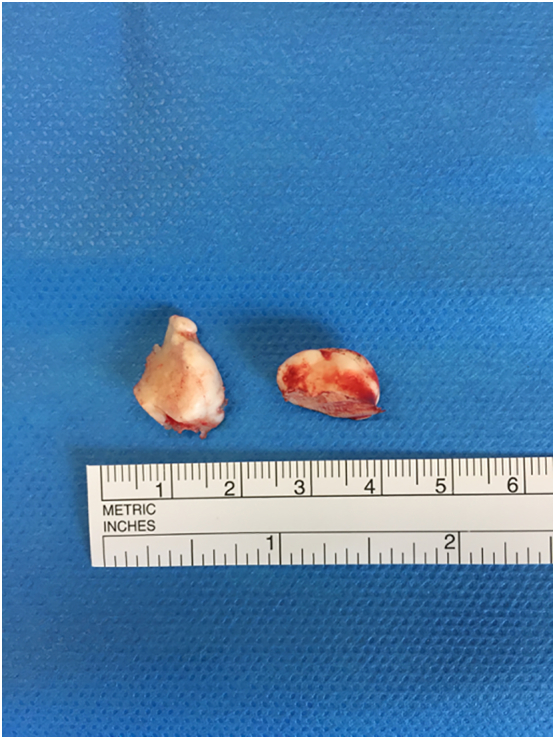
Upper view and size of the mandibular tori.

Histological analysis found mature cortical bone tissue with no cellular atypia. The two tori measured 1.67 cm and 1.16 cm maximum thickness, with full thickness excision (Fig. 3).

At one month of follow-up, a local recurrence with a similar volume as initially (+/− 0.1 cm) was found in the same area. A CT scan was performed to confirm the new recurrence. Considering patient discomfort, a second surgery was carried out (same procedure as the first surgery), but we prescribed a mouth guard, made by our prosthetist, in the postoperative period.

Histological analysis of the recurrence revealed a nodular cortical bone structure with very small caliber interosseous spaces containing capillary vessels. The second analysis was similar to the first, confirming recurrence.

**Fig. 4:**
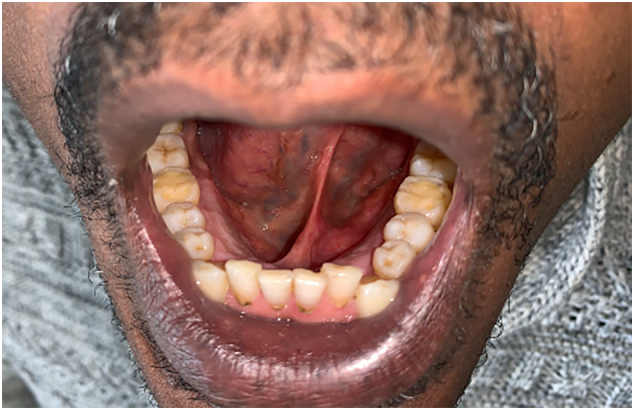
Result at one year postoperatively.

Follow-up one year after the second surgery found no recurrence, and the patient was satisfied with the outcome (Fig. 4).

## 3 Discussion

This article describes a rare case of early recurrence of mandibular torus after surgical resection which may have been induced by mechanical stress.

Rodríguez-Vázquez and al [7], in studies on fetuses, introduced a theory according to which there is a constant bone prominence at birth, which varies in size and shape in relation to the mental foramen. This bony prominence, at the origin of the mandibular torus, is formed when the mandibular internal lamina reaches the posterior and upper part of the Meckel cartilage during fetal development.

From a genetic point of view, the palatal torus may be hereditary, with autosomal dominant inheritance and a penetrance of 85% [5;8]. On the contrary, the mandibular torus appears to be less influenced by genetics. An allele on the X chromosome may be normal or predisposing to palatal torus, which would explain the presence of these exostoses mainly in women [10].

The incidence of tori depends on the population, with higher frequencies in Caucasian and Asian populations [10]. Palatal tori tend to affect females between the 3rd and 4th decade of life, while mandibular tori mostly affect males in the 4th decade [5,10].

.

Trauma seems to be an inducing factor [6]. Mandibular tori are most frequent near the mental foramen, which is a mandibular weak point where the majority of the mechanical stress is exerted. Excessive mechanical stress is responsible for the transduction signal for osteoblasts, favoring the formation of exostoses [10].

.These mechanical stresses can be found in individuals with bruxism or with excessive consumption of hard foods (as in Viking populations [11]), or in patients with dental prosthesis contact [1;5–10].

Finally, various dietary factors have been shown to be associated with the occurrence of torus, notably saltwater fish. These fish are rich in polyunsaturated fatty acids and vitamin D, which have a role in calcium and phosphate regulation and therefore in bone development. This is also the case with excessive consumption of calcium-rich products [10].

Our patient presented several risk factors for torus development: male sex, ethnic origin, heavy consumption of saltwater fish for a large part of his life (rich in vitamin D and polyunsaturated fatty acids), and bruxism. Bruxism is a likely explanation for the early recurrence in this case seeing as there was no further recurrence when the patient was using a mouth guard.

## 4 Conclusion

The presence of a torus can be disabling for a patient. Therefore, during management, the surgeon should try to avoid recurrence by taking into account the potential factors of risk. This case underlines the importance of mechanical overload, which may be responsible for the recurrence of torus, particularly when associated with dietary and ethnic factors. The detection of the factors favoring recurrence is a major challenge for surgeons.

## Ethical approval

The ethical committee of the hospital gave agreement to report this case. The writing was performed according to the rules of anonymity.

## Funding

The author do not declare any financial sources in the production of this article.

## Author contribution

Dr. Rabuel Valentin: wrote this article, data collection, perform the surgery.

Dr. Levasseur Julie: Data collection, perform the surgery too.

Dr. Moris Vivien and Dr Guillier David: reviewed the article and checked the scientific sources.

Dr. Gengler Charline: Help for the translation of this article.

Pr Zwetyenga Narcisse: Head of department who supervised the writing of the manuscript.

## Consent

Written informed consent was obtained from the patient for publication of this case report and accompanying images. A copy of the written consent is available for review by the Editor-in-Chief of this journal on request.

## Guarantor

Valentin Rabuel is the guarantor and accepts full responsibility for the work and/or the conduct of the study, had access to the data, and controlled the decision to publish.

## Research registration number

Not applicable.

## Provenance and peer review

Not commissioned, externally peer-reviewed.

## Declaration of competing interest

The authors state that they have no conflicts of interest for this report.

## References

[1] Chossegros C., Foletti J.M., Graillon N., Guyot L. (2016). Les Torus ou Torii de la cavité buccale, pourquoi?. Revue de Stomatologie, de Chirurgie Maxillo-faciale et de Chirurgie Orale.

[2] García-García Andrés S., Martínez-González José-María, Gómez-Font Rafael, Soto-Rivadeneira Angeles, Oviedo-Roldán Lucia (2010). Medicina Oral, Patologia Oral Y Cirugia Bucal.

[3] Flores Meza, Luis José (2004). Torus palatinus and torus mandibularis. Revista De Gastroenterologia Del Peru: Organo Oficial De La Sociedad De Gastroenterologia Del Peru.

[4] Rouas A., Midy D. (1997). About a mandibular hyperostosis: the torus mandibularis. Surg. Radiol. Anat..

[5] Loukas M., Hulsberg P., Tubbs R.S., Kapos T., Wartmann C.T., Shaffer K. (2013). The tori of the mouth and ear: a review. Clin. Anat..

[6] Cortes Arthur Rodriguez Gonzalez, Jin Zhaoyu, Morrison Matthew Daniel, Arita Emiko Saito, Song Jun, Tamimi Faleh (2014). Mandibular tori are associated with mechanical stress and mandibular shape. J. Oral Maxillofac. Surg..

[7] Rodríguez-Vázquez José Francisco, Sakiyama Koji, Verdugo-López Samuel, Amano Osamu, Murakami Gen, Abe Shinichi (2013). Origin of the torus mandibularis: an embryological hypothesis. Clinical Anatomy (New York, N.Y.).

[8] Auskalnis A., Bernhardt O., Putniene E., Sidlauskas A., Andriuskeviciute I., Baseviciene N. (2015). Oral bony outgrowths: prevalence and genetic factor influence. Study of twins. Medicina (Kaunas).

[9] Agha R.A., Franchi T., Sohrabi C., Mathew G., Kerwan A. (2020). SCARE Group, TheSCARE 2020 guideline: updating consensus Surgical CAse REport (SCARE)guidelines. Int. J. Surg..

[10] Hascoet Emilie, Vaillant Pierre Yves, Tempescul Adrian, Darbin Caroline, Lansonneur Cedric, Boisramé Sylvie (2015). Tori et exostoses multiples: présentation d’un cas et revue de la littérature. Médecine Buccale Chirurgie Buccale.

[11] Richter S., Eliasson S.T. (2012). Prevalence of torus mandibularis in Viking Age Icelanders. Bull. Int. Assoc. Paleodont..

